# Effects of an e-Learning Program (Physiotherapy Exercise and Physical Activity for Knee Osteoarthritis [PEAK]) on Chinese Physical Therapists’ Confidence and Knowledge: Randomized Controlled Trial

**DOI:** 10.2196/71057

**Published:** 2025-04-18

**Authors:** Zi-Ru Wang, Yunqi Wang, Shuning Duan, Xier Chen, Guoxin Ni

**Affiliations:** 1 School of Sport Medicine and Rehabilitation Beijing Sport University Beijing China; 2 School of Physical Education and Health Longyan University Longyan China; 3 School of Sports Medicine Wuhan Sports University Wuhan China; 4 Department of Rehabilitation Medicine School of Medicine, Xiamen University First Affiliated Hospital of Xiamen University Xiamen China

**Keywords:** osteoarthritis, telehealth, exercise therapy, e-learning, physiotherapist, Knee Osteoarthritis Knowledge Scale

## Abstract

**Background:**

Knee osteoarthritis (OA) presents a significant burden in China due to its high prevalence, aging population, and rising obesity rates. Despite clinical guidelines recommending evidence-based care, limited practitioner training and inadequate telehealth integration hinder effective OA management.

**Objective:**

The aim of this study was to evaluate the effectiveness of an e-learning program in improving the confidence and knowledge of Chinese physical therapists in managing knee OA and to explore their perceptions of the program.

**Methods:**

This was a randomized controlled trial with 2 parallel arms involving 81 rehabilitation practitioners from 18 Chinese provinces. The intervention group completed a 4-week web-based training program (Physiotherapy Exercise and Physical Activity for Knee Osteoarthritis [PEAK]-Chinese), while the control group received no training. The primary outcome was self-reported confidence in OA management (11-point scale). Secondary outcomes included knowledge (Chinese Knee Osteoarthritis Knowledge Scale [KOAKS]) and likelihood of clinical application of core OA treatments. Process measures and semistructured interviews captured participants’ training perceptions. Quantitative data were analyzed using regression models, 2-sided *t* tests, and descriptive statistics, while thematic analysis was performed on the interview data of 10 participants.

**Results:**

A total of 80 participants completed the outcome measures at 4 weeks. The intervention group demonstrated significant improvements in confidence compared to the control group, including managing OA with exercise-based programs (adjusted mean difference=3.27, 95% CI 2.72-3.81), prescribing exercise (adjusted mean difference=3.13, 95% CI 2.55-3.72), and delivering telehealth (adjusted mean difference=4.41, 95% CI 3.77-5.05). KOAKS scores also improved significantly (mean change=9.46); however, certain belief bias related to OA concepts and the use of scans remained unchanged (25/41, 61% and 27/41, 66%, respectively). Approximately 73% (30/41) of the intervention participants rated the course as extremely useful. Interviews emphasized the need for cultural adaptation and practical telehealth training with real-life scenarios to enhance program applicability.

**Conclusions:**

The PEAK program improved Chinese practitioners’ confidence and knowledge in managing knee OA, underscoring e-learning’s potential to support evidence-based OA care in China. To optimize future implementations, further research strategies could include enhancing cultural relevance, addressing misconceptions, and incorporating practical, real-world training.

**Trial Registration:**

Chinese Clinical Trial Register ChiCTR2400091007; https://www.chictr.org.cn/showproj.html?proj=239680

## Introduction

Osteoarthritis (OA) is a multifactorial joint disease that is highly prevalent worldwide, affecting 133 million people in China as of 2019 [1]. The burden of OA in China is projected to continue rising until 2044 due to the aging population, rising obesity rates, and the country’s large population size [2,3]. The synthesis of 11 high-quality clinical practice guidelines for OA management recommends exercise, patient education, and weight management as first-line treatments before considering injections, drugs, or surgery [4], as they have demonstrated benefits on joint pain, physical function, quality of life, and delaying or preventing the surgery [5-8]. However, the uptake of this guideline-based care remains suboptimal, and a series of disconnects exist between all the potential key stakeholders [9], with barriers to implementation occurring at multiple levels [10,11]. Specifically, synthesizing evidence [12] highlights that practitioners often face knowledge gaps and lack effective communication and behavior change skills. Addressing these gaps through training and education has been identified as a critical implementation strategy [13].

The first assessor may play a central role in managing people with OA, and physiotherapist-led care may be better than a doctor visit for enacting clinical guidelines [14]. However, in China, unlike in other countries where OA is primarily managed in primary care settings, individuals can directly select any health care professional as their first contact without referrals [15]. Further, the role of physiotherapists in China is still unstructured and remains in the developing stages to date, with a persistent shortage of trained specialists [16,17]. In addition to workforce limitations and the lack of knowledge and skills among health care professionals, other challenges such as inequity and unaffordability of care and fragmented health care services have been highlighted in the implementation of core OA treatments in many middle-income countries. [18]. Telehealth offers a potential solution to these barriers and is becoming a popular approach to address the unequal allocation of health care resources in China [19], with the market projected to reach US $523.10 million in revenue by 2030 [20]. Growing evidence has supported its effectiveness in OA management [21]; specifically, videoconference-based telehealth has been shown to effectively deliver exercise therapy for people with OA [22-24]. This further warrants the target training for health care professionals, as a recent study [25] highlighted Chinese physicians’ need for telehealth communication skills in chronic disease management. Therefore, to address the growing demand for high-quality OA care in China, providing training interventions to upskill physiotherapists and rehabilitation specialists [26] in both knowledge of OA and telehealth is critical.

The Physiotherapy Exercise and Physical Activity for Knee Osteoarthritis (PEAK) program [27] is a structured e-learning program for best-practice knee OA management. It guides physiotherapists in delivering a protocolized management program focused on education, exercise, and physical activity over 5 one-to-one consultations via videoconferencing. The PEAK trial protocol details the main components of each consultation and intervention [28]. This program has demonstrated clinical implementation effectiveness in Australia through a noninferiority trial [29] and has provided outcomes comparable to those seen in in-person care. It was also perceived as effective, acceptable, and useful by participants [30,31]. Thus, this study aims to (1) evaluate the effects of the PEAK e-learning program on building Chinese physical therapists’ confidence in delivering core recommended OA treatments via telehealth and improving their knowledge of evidence-based OA practices and (2) investigate their perspectives on this educational intervention.

## Methods

### Study Design

This study is a web-based randomized controlled trial with 2 parallel arms. The trial is registered with the Chinese Clinical Trial Registry (ChiCTR2400091007). This is a fully web-based trial with web-based recruitment, intervention, management, and assessment. This study adheres to the CONSORT (Consolidated Standards of Reporting Trials) guidelines [32] (Multimedia Appendix 1). Study participants provided digital informed consent through a web-based recruitment survey.

### Ethics Approval

Ethics approval for this study was granted by the ethics committee of the First Affiliated Hospital of Xiamen University (approval XMFHIIT-2023SL140) in accordance with institutional guidelines. Informed consent was obtained from all the participants, and data were anonymized to ensure privacy and confidentiality. No compensation was provided for participation.

### Participants and Sample Size

Participants were practitioners who met the following inclusion criteria: (1) currently engaged in clinical practice related to OA management, (2) completed professional clinical education or obtained a relevant practice qualification in rehabilitation or physical therapy, (3) had access to a computer with an internet connection, and (4) were willing to complete a 4-week web-based training program (if allocated to the intervention group) and refrain from participating in other professional development activities during the study period.

Participants were recruited from various clinical settings across China through digital flyers distributed on WeChat. A target sample size of 80 participants was determined, accounting for an anticipated dropout rate of 35% and a large effect size (0.8). This estimation was based on data from similar trials [30,33,34] evaluating the impact of web-based arthritis-related training programs on physical therapists’ confidence.

### Randomization Procedures

After a researcher (YW) screened the eligibility criteria online and completed the enrollment process, participants were randomly assigned to either the intervention group (PEAK-Chinese training) or the control group (no training) with an allocation ratio 1:1. Randomization was conducted using a computer-generated sequence with a predefined randomization seed by a blind researcher (XC). Stratification was performed based on baseline objective knowledge levels measured by Knee Osteoarthritis Knowledge Scale (KOAKS) scores [35], as it has higher reliability and objectivity compared to other self-reported outcome measures. This approach ensured that participants with varying initial OA knowledge levels were evenly distributed between groups, thereby reducing potential confounding effects and improving the validity of between-group comparisons. To maintain balance within each knowledge level, participants with low baseline knowledge (KOAKS below median score) were randomized in blocks of 6 and those with high baseline knowledge (KOAKS above median score) were randomized in blocks of 4. The larger block size for the lower-knowledge group was chosen to better distribute the potential variability in the learning effects, while the smaller block size in the higher-knowledge group ensured precision in balancing the experienced learners. Within each stratum, equal numbers of participants were allocated to the intervention and control groups, ensuring balance across baseline knowledge levels and minimizing the potential confounding factors.

This was a single-blind study, as complete blinding of participants was not feasible due to the nature of the intervention. To minimize bias in data collection and analysis, the statistician responsible for quantitative data analysis remained blinded to group allocation, and all data were deidentified prior to analysis. Audio recordings and transcripts of the interviews were deidentified by a researcher (YW) who was not involved in the thematic analysis process to prevent potential interpretative biases.

### Intervention

The original PEAK program, developed in English, was translated into simplified Chinese, including all the e-learning components such as textual content, embedded infographics, downloadable booklet resources (preparing for your consultations, OA information, exercise booklet, knee plan and log book, Zoom troubleshoot guide, initiating and using Zoom for video consultations, accessing the website of exercise videos, preconsultation survey, consultation outline, and readiness checklist), and Chinese transcripts and subtitles of the embedded videos. Some components were further adjusted to adapt the Chinese context. For instance, the technical instructions for using the Zoom platform, which is not widely accessible in China, were supplemented with guidance on Tencent Meeting, a commonly used videoconferencing platform. The Chinese version of the PEAK program consists of structured e-learning modules covering evidence-based OA management, remote physiotherapy practices, and clinical application of the PEAK physiotherapy treatment protocol. Each module includes video demonstrations, interactive quizzes, and downloadable resources such as exercise manuals and patient education materials. The translated program was launched on a web-based platform [36], making it accessible to participants within their local network.

Participants in the intervention group completed the free PEAK-Chinese training program over 4 weeks. To monitor participants’ learning progress, we used a localized web-based study management platform (Xiaoetong). This platform provides information about the training program, registration and learning instructions, and an organized module directory. Participants were required to upload screenshots of their module completion status to the platform as evidence of progress. Research staff monitored and reviewed participant progress through the platform. Additionally, a WeChat learning group was established and managed by research assistants not involved in data analysis to provide technical support and facilitate learning discussions. Participants were deemed to have completed the training if they demonstrated at least 90% progress and submitted screenshots confirming the completion of all quizzes embedded within the program. Participants in the control group did not receive any training during the study period.

### Data Collection and Measurements

#### Demographic Information

The demographic information of all the participants was collected at baseline, including age, gender, highest educational level, profession, years of experience in OA-related clinical practice, primary city of clinical practice, OA-related professional certification, and prior participation in OA-related training programs.

#### Primary Outcome

The primary outcome was participants’ self-reported confidence in managing knee OA by using exercise-based programs. Confidence was assessed using an 11-point numeric rating scale, where 0 indicated “not confident at all” and 10 represented “extremely confident.” Two domains of confidence were measured: confidence in applying exercise knowledge to manage patients and confidence in designing strength training programs for patients. These single-item confidence measures were adapted from a real-world evaluation study [30] of the original English PEAK program, which has previously used similar self-reported ratings to assess training effectiveness.

#### Secondary Outcomes

##### Objective Knowledge Level

The objective knowledge of knee OA was assessed using the Chinese version of KOAKS [35], an 11-item instrument that evaluates knowledge across 5 domains: causation, diagnosis, symptom interpretation, management principles, and treatment and self-care options. The scale has already been psychometrically validated as reliable for Chinese respondents in a previous study [35]. Each item was rated on a 5-point Likert scale, with reverse scoring applied to specific items to address misconceptions. Total scores ranged from 11 to 55, with higher scores indicating greater knowledge.

##### Confidence in Telehealth Delivery

Participants’ confidence in delivering care via telehealth platforms was assessed using the same 11-point numeric rating scale as the primary outcomes, with higher scores indicating greater self-reported confidence level.

##### Likelihood of Clinical Application

Participants rated the likelihood of integrating evidence-based OA management strategies into their clinical practice, including patient education, prescribing individualized strength training regimens, and recommending physical activity programs. Likelihood ratings were assessed using the same 11-point numeric rating scale, with higher scores indicating a greater likelihood of application.

### Process Measures

Process measures were collected exclusively from the intervention group to evaluate participants’ perceptions of the training program. These included ratings of the overall usefulness of the course content, utility of the downloadable resources, value of the exercise video library, and the course’s usefulness on developing their telehealth and web-based consultation skills. These measures were assessed using a 4-point Likert scale ranging from “1=not useful at all” to “4=extremely useful.”

### Qualitative Feedback

To complement the quantitative findings, semistructured interviews were conducted with participants from the intervention group following the completion of the training. The interviews aimed to explore participants’ training experiences, perceived effectiveness, barriers to implementation, and self-efficacy in applying the learned skills. The interview guide was adapted from the original PEAK qualitative study [31]. Additionally, the interviews specifically sought to identify potential areas for improvement and enhancement of the training intervention to support the future development of a comprehensive OA model of care for Chinese health care professionals.

### Data Collection

The measurement survey items are detailed in Multimedia Appendix 2. Outcome data were collected at baseline and at 4 weeks after the intervention by using web-based questionnaires administered via Wenjuanxing, a widely used web-based survey platform in China that allows users to design, distribute, and analyze questionnaires. Semistructured interviews were conducted by a researcher (YW) trained in qualitative research within 2 weeks after training completion to capture participants’ recent experiences and reflections on the program. Interviews were conducted online via voice call (WeChat or Tencent videoconference call), lasting approximately 20 minutes each.

### Statistical Analysis

Quantitative data were analyzed using linear regression models adjusted for baseline scores to estimate between-group differences in change scores for outcomes about confidence level and likelihood of clinical application, with results reported as adjusted mean differences and 95% CIs. Model assumptions, including normality and homoscedasticity, were assessed using diagnostic plots. Group differences in KOAKS scores were assessed using independent sample 2-sided *t* tests, assuming normally distributed residuals. Descriptive statistics were calculated for process measures. Process measures were described in mean (SD) or number (frequency). A 2-tailed *P* value <.05 was considered statistically significant for all tests.

Thematic analysis was conducted to identify recurring themes and subthemes from the interview data. Transcripts were read multiple times by 2 researchers (SD and XC) to ensure familiarity with the data and to independently code text into preliminary topics. Discrepancies in coding were resolved through iterative discussions to reach consensus, and related topics were grouped collaboratively to develop overarching themes. A third researcher (Wang ZR), experienced in qualitative analysis and uninvolved in the interviews, reviewed all the transcripts to validate the consistency and relevance of the refined emergent themes. Illustrative quotes were included in the results to provide transparency and demonstrate the depth of participant experiences. All statistical analyses were performed using R software (version 4.2.0, R Core Team) for quantitative data and NVivo software (version 12, Lumivero) for qualitative data.

## Results

### Characteristics of the Participants

**Figure 1:**
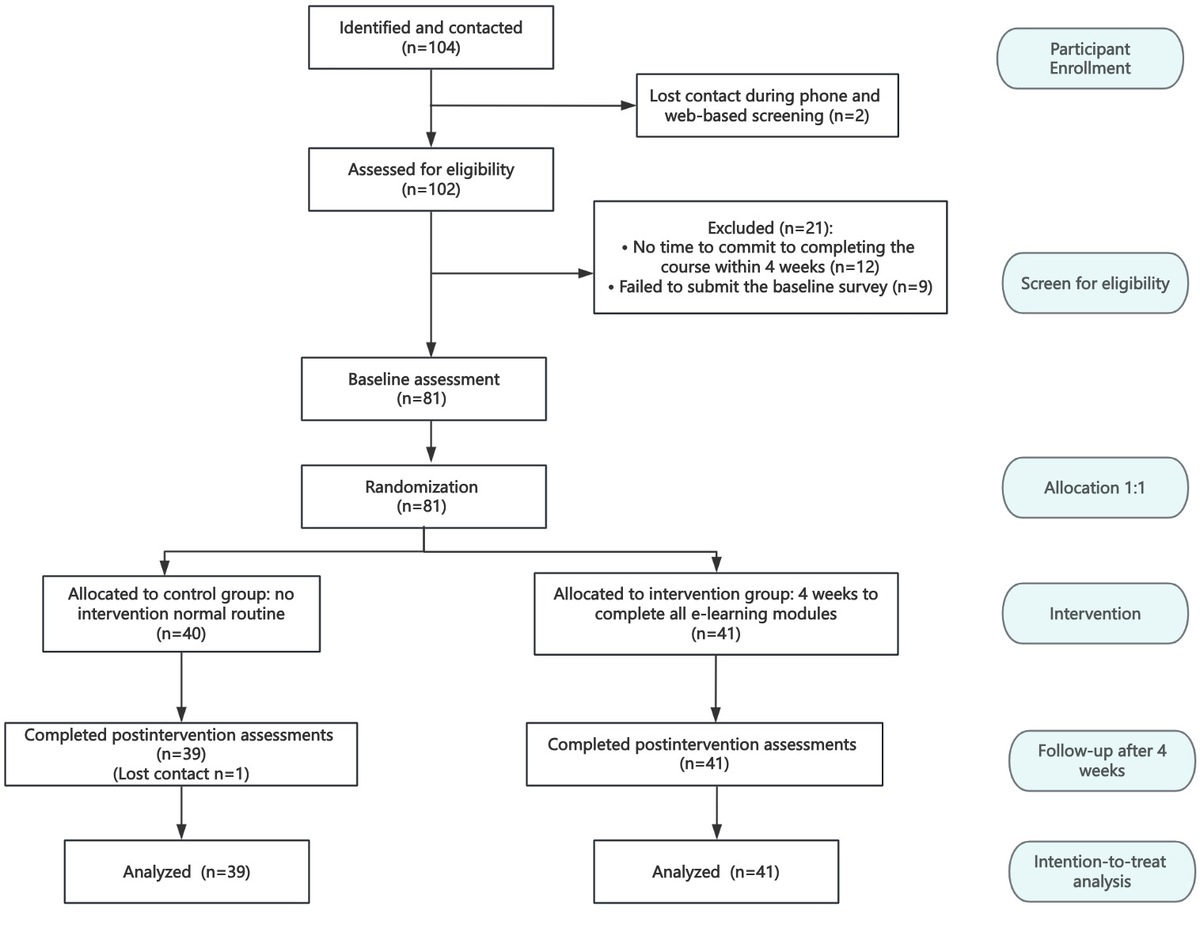
Flowchart in this study.

A total of 81 eligible participants from 18 provinces in China completed the baseline measures between January and February 2024. Following the randomization process, 41 participants were allocated to the intervention group and 40 to the control group. A total of 80 participants completed the postintervention (4-week) follow-up measurements. Figure 1 presents the detailed flow diagram of this study, and the demographic characteristics of the participants are summarized in Table 1.

**Table 1:** Demographic characteristics of the whole group (N=81).

Characteristic		Values	
Age (years), mean (SD)		27.8 (5.37)	
**Gender, n (%)**			
	Female		51 (63)
	Male		30 (37)
**Education level, n (%)**			
	Bachelor’s degree		64 (79)
	College diploma		11 (14)
	Graduate degree		6 (7)
**Professional background, n (%)**			
	Rehabilitation medicine/physiotherapy		67 (83)
	Sports medicine		8 (10)
	Exercise and fitness practitioners (nonmedical)		4 (5)
	Rehabilitation practitioners with traditional Chinese medicine background		2 (2)
**Years of clinical experience, n (%)**			
	<1 year (includes interns)		57 (70)
	1-5 years		18 (22)
	6-10 years		4 (5)
	>10 years		2 (3)
**Relevant qualification, n (%)**			
	Yes		71 (88)
	No		10 (12)
**Previous osteoarthritis training, n (%)**			
	Yes		4 (5)
	No		77 (95)

### Primary Outcomes

After a 4-week PEAK-Chinese e-learning, the intervention group demonstrated significant improvements in self-reported confidence levels compared to the control group (Table 2) across both knowledge about exercise to manage patients with knee OA (Cohen *d*=2.35) and their ability to prescribe a strengthening exercise program for patients with knee OA (Cohen *d*=2.23). The effect sizes were large, indicating a strong and clinically meaningful impact of the intervention on participants’ confidence in applying exercise knowledge and skills learned from the program. Additionally, baseline confidence levels were found to significantly contribute (*P*<.001) to both the observed changes, highlighting the importance of accounting for baseline variability in the analysis. These results suggest that the intervention effectively enhanced participants’ confidence irrespective of the initial baseline levels.

**Table 2:** Outcomes at different timepoints and differences in the change between the groups.

	Baseline, mean (SD)		At 4 weeks, mean (SD)		Change between groups^a^
	Intervention (n=41)	Control (n=40)	Intervention (n=41)	Control (n=39)	Adjusted mean difference (95% CI)
Confidence in exercise knowledge	5.00 (1.34)	4.77 (1.51)	8.71 (0.98)	5.33 (1.72)	3.27 (2.72-3.81)
Confidence in prescribing exercise	4.68 (1.42)	4.92 (1.78)	8.85 (0.96)	5.79 (1.75)	3.13 (2.55-3.72)
Confidence in videoconferencing	3.37 (1.55)	3.18 (1.60)	8.49 (1.12)	4.03 (1.81)	4.41 (3.77-5.05)
KOAKS^b^ score	36.52 (5.75)	35.85 (5.00)	47.17 (5.22)	37.44 (5.48)	9.46 (7.50-11.42)
Likelihood of using patient education	5.88 (1.66)	5.95 (1.65)	9.20 (0.90)	6.79 (1.59)	2.42 (1.87-2.97)
Likelihood of using strengthening exercise	5.78 (1.82)	6.10 (1.79)	9.34 (0.86)	6.85 (1.29)	2.56 (2.11-3.02)
Likelihood of using physical activity	3.83 (1.40)	3.85 (1.42)	8.98 (1.15)	5.03 (1.41)	3.96 (3.46-4.46)

^a^*P*<.001, meaning there was a significant intervention effect across the 2 groups.

^b^KOAKS: Knee Osteoarthritis Knowledge Scale.

### Secondary Outcomes

#### KOAKS Change Score

KOAKS scores were balanced at baseline between the intervention and control groups due to stratified randomization (t_78_=0.23; *P*=.82). Normality assumptions for KOAKS change scores were met based on Shapiro-Wilk tests (*P*>.05 for both groups). Therefore, an independent sample 2-sided *t* test was conducted to compare KOAKS change scores between the 2 groups. Results showed that the intervention group had a significantly greater improvement (t_78_=9.53; *P*<.001; Cohen *d*=2.13) in objective overall knowledge level of knee OA compared to the control group (see Table 2). However, for participants following the PEAK training, the scores on some specific items remained lower, particularly regarding knowledge of OA concepts and the use or role of radiographies or scans. Specifically, after the intervention, 61% (25/41) of the participants still believed that joints wear out with everyday use, and only 34% (14/41) understood that a radiograph is not necessary for diagnosing OA. This may indicate that the relevant content in the program (modules in PEAK week 2) did not effectively address or change certain misconceptions. The mean score for each of the 11 items in the intervention group is presented in Table 3.

**Table 3:** Mean scores of each Knee Osteoarthritis Knowledge Scale item in the intervention group post intervention.

Knee Osteoarthritis Knowledge Scale item	Values, mean (SD)
1. Your knee joint wears out with everyday use	2.41 (1.84)
2. Osteoarthritis will only get worse over time	4.10 (1.30)
3. Increased knee pain always means that you have damaged your knee	4.02 (0.79)
4. You need a radiograph or scan to know if you have osteoarthritis	3.32 (1.31)
5. Being active makes osteoarthritis feel better	4.85 (0.42)
6. Keeping a healthy body weight is a key part of osteoarthritis care	4.90 (0.30)
7. Radiographs or scans show how much your osteoarthritis affects you	3.80 (1.01)
8. Making your leg muscles stronger improves your ability to do daily tasks	4.95 (0.22)
9. Pain from osteoarthritis can be managed without surgery	4.78 (0.48)
10. Exercises can ease pain as much as most medications	4.68 (0.61)
11. Most people with knee osteoarthritis will need a joint replacement at some point	4.61 (0.67)

#### Confidence in Delivering OA Care Via Telehealth

Regression analysis demonstrated a significant improvement (Table 2) in participants’ confidence in delivering OA care through web-based consultations using videoconferencing platforms or other telehealth tools (such as WeChat, a commonly used multifunctional digital application in China), with a large effect size (Cohen *d*=2.95).

#### Likelihood of Clinical Application of Core OA Treatments

**Figure 2:**
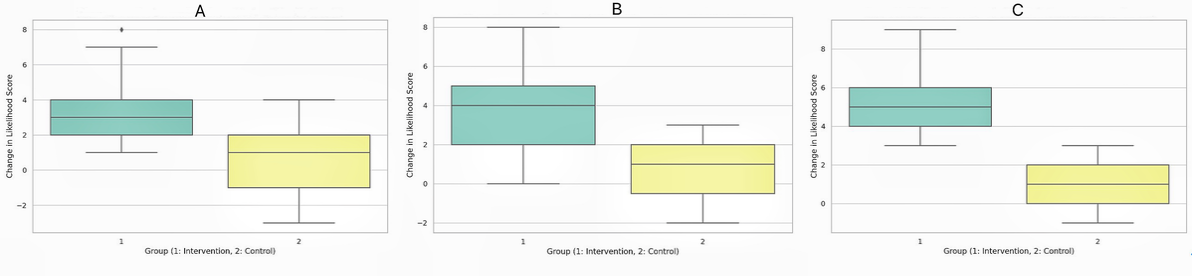
Boxplots showing the change scores for the likelihood to use core osteoarthritis treatments across groups. (A) Education. (B) Strengthening exercise. (C) Physical activity.

As shown in Table 2, participants demonstrated significant improvements after the PEAK training in the likelihood of incorporating all the first-line core treatments, including patient education (Cohen *d*=1.89), strength training plans (Cohen *d*=2.35), and physical activity plans (Cohen *d*=3.09) into their real-world clinical practice. The boxplots in Figure 2 show the change scores for the likelihood of using these core treatments, where the intervention group consistently demonstrated higher median scores compared to the control group, with narrower interquartile ranges indicating less variability.

#### Process Evaluation

**Figure 3:**
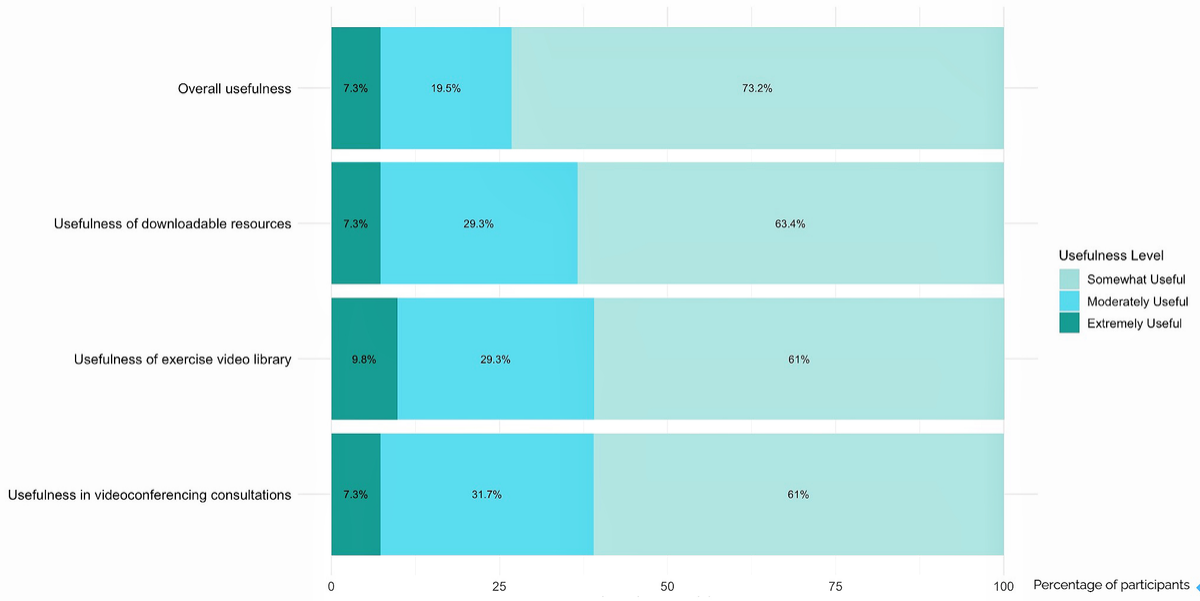
Perceptions of the usefulness of course resources.

All participants in the intervention group (n=41) completed the training (achieving >90% progress in the PEAK program) and the follow-up measures. Participants rated the course as highly useful, with mean scores across the 4 evaluated aspects all exceeding 3.5 on a 4-point Likert scale—with the highest mean score for the overall PEAK contents (mean 3.66, SD 0.617), followed by the course’s support for videoconferencing consultations (mean 3.54, SD 0.636) and the usefulness of the exercise video library. As shown in Figure 3, all participants in the intervention group found the PEAK course useful, with more than half rating it as extremely useful across all components (eg, overall course, downloadable resources, exercise videos, videoconferencing consultations). These findings highlight consistently positive perceptions of the course materials and tools among participants who completed this e-learning program.

#### Potential Moderator

When each of the potential moderators (baseline KOAKS scores, age, gender, location of clinical setting, time in practice, education level, and professional certification) was included as a covariate in the regression models, none of these variables were significant contributors to explaining the changes in confidence or the likelihood of using core treatments (*P*>.05). These findings suggest that the intervention’s effects on improving these outcomes were consistent, regardless of the baseline OA knowledge levels or demographic characteristics of the participants in this study.

### Emerged Themes From Semistructured Interviews

Following the completion of the PEAK e-learning, 10 participants from the intervention group were interviewed to share their feedback on their learning process. The thematic analysis of the interview data revealed 4 key themes, each addressing different aspects of participants’ experiences and perceptions of the PEAK training program.

#### Theme 1: Self-Efficacy and Initial Perceptions

Participants entered the training with varying levels of confidence in their clinical abilities, influenced by limited exposure to telehealth and an evidence-based understanding of OA management. Many participants described their previous clinical rehabilitation practices as rooted in manual therapy and conventional face-to-face workflows, which provided little preparation for telehealth. One participant described their routine as “mostly manual therapy and physical on hand treatments, rarely involving remote care.”

For telehealth, participants expressed a lack of familiarity, with some exposure limited to social media platforms rather than professional training. As one participant noted, “I didn’t have any prior experience with telehealth—just saw some related videos on TikTok, which felt more casual than professional.” This lack of structured exposure led to low initial confidence in their ability to effectively deliver OA management remotely.

#### Theme 2: Relevance and Accessibility of Training Content

The cultural and practical relevance of the training materials emerged as a critical concern. Participants highlighted that the predominantly non–Asian-focused images and scenarios in the modules created a perceived disconnect with their predominantly Asian patient population. A participant noted, “All the materials—videos and images—featured White patients, which felt less relatable for our local context.”

Another point of feedback concerned the delivery format—with text-heavy modules being perceived as less engaging compared to multimedia formats such as videos and interactive modules. One participant suggested, “Adding more videos and fewer lengthy texts would make the learning process more engaging and easier to understand.”

#### Theme 3: Challenges to Telehealth Implementation

The challenges of integrating telehealth into clinical practice emerged as a recurring theme. Although participants acknowledged the potential of telehealth to expand access to care, they expressed concerns about patient engagement and perceived value. A common issue was patients questioning the cost-effectiveness of remote care compared to traditional in-person consultations. One participant shared, “Patients think that web-based consultations should be cheaper or might not work as effectively.”

Technological barriers of videoconferencing also posed challenges, particularly among older patients or retirees in China due to the lack of familiarity. In contrast, WeChat was described as more accessible, given their ease of use among all Chinese individuals. As one participant shared, “WeChat video calls are something everyone already knows how to use, and there’s no learning barrier for the patients.” However, concerns were raised about the informal nature of using personal accounts for professional consultations.

#### Theme 4: Practical Impact on Future Clinical Practice

Participants acknowledged the potential of the training program to influence their clinical approach. Some described shifts in their understanding of OA management, particularly regarding the relationship between pain and exercise. As one participant noted, “I used to think pain meant damage, but now I understand it’s much more complex, and that is the timepoint I start to shift my beliefs on exercise therapy.”

The training offered foundational knowledge and insights into OA management. However, participants noted that their learning needs extended beyond theoretical frameworks to practical applications, especially in telehealth scenarios. Many favored in-person training for its ability to provide real-time feedback. One participant remarked, “The training clarified key concepts about OA management but lacked direct guidance and real-life practices on how to deliver telehealth consultations.”

## Discussion

### Principal Findings

This study employs a web-based randomized controlled design with a mixed-method evaluation to comprehensively assess the impact of PEAK-Chinese version program on Chinese physiotherapists’ confidence, knowledge, perceived usefulness, application likelihood, and suggestions for program improvement. Our findings revealed that the intervention group demonstrated significantly greater improvements across all 6 outcomes compared to the control group, and the usefulness of the learning content was highly rated by the participants. Moreover, the improvements observed in the PEAK-Chinese intervention were notably greater in magnitude and scope compared to those reported in research using international data from the original PEAK program [30]. Although both programs significantly enhanced participants’ confidence and likelihood of applying key OA treatments, the effect sizes in PEAK-Chinese (Cohen *d*=1.89-3.09) were substantially larger than those reported in PEAK-English (*d*=0.27-1.46). Specifically, confidence in videoconferencing improved more in PEAK-Chinese (*d*=2.95) than that in PEAK-English (*d*=1.29), suggesting a stronger impact of the intervention in a population with lower baseline familiarity with telehealth. This may further encourage the use of other digital health tools to support exercise management. For example, increasing evidence supports the effectiveness of virtual tools in improving knee OA management [37], including cell phone, text messages, website, mobile apps, and wearable devices [38]. Similarly, the likelihood of implementing OA treatments such as patient education (*d*=1.89 vs 0.27), strengthening exercises (*d*=2.35 vs 0.31), and physical activity (*d*=3.09 vs 0.44) showed markedly larger improvements in the Chinese cohort. The PEAK-Chinese program appears to have had a more transformative impact, likely due to greater initial disparities in knowledge and confidence among our learners, reinforcing the importance of such context-specific and culturally adapted e-learning interventions for high value OA management.

Certain misconceptions about OA (eg, OA is wear and tear with every use) were identified in our study through both quantitative and qualitative methods and were found to be difficult to change, even after the intervention. This widespread misunderstanding of OA management is a global issue [39], often leading to fear-avoidance behaviors toward exercise and hindering patients from achieving favorable prognoses [40]. To facilitate this conceptual shift in understanding OA, health care professionals are recommended to change the information, shift the focus of treatment, and involve patients in the decision-making process [41]. It is important to emphasize a whole-person enactive perspective [42] rather than a disease-focused approach to empower patients with chronic pain to make positive behavior change [43]. Thus, future behavior change interventions for Chinese practitioners should emphasize the development of transdisciplinary core capabilities [44] to enhance person-centered approaches to achieve long-term health outcomes. Most participants emphasized the importance of scans, likely due to their routine availability in Chinese public hospitals without appointments or long waits. This convenience, however, increases reliance on imaging and burdens radiologists [45], highlighting the need for health economic evaluations to guide long-term decisions. Notably, all participants in this study held specialist certifications, but these qualifications did not authorize them to independently prescribe exercise plans for the patients [46]. The current training system for physiotherapists in China requires further improvements, as critical responsibilities such as prescribing exercise programs remain primarily under physicians rather than physiotherapists themselves [47], where this dynamic may perpetuate an impairment-based fixing narrative [43]. Changing rooted misbeliefs requires substantial effort and resources; therefore, it is particularly important to cultivate correct evidence-based beliefs in the early stage [48]. This further underscores the need for systemic changes to professional rehabilitation curricula to facilitate the implementation of a person-centered approach and to emphasize the professional roles of rehabilitation practitioners in managing individuals with OA [49]. Although demographic factors were not significant predictors in the regression model for outcome variables (eg, confidence in using exercise therapy) in this study, future research could explore potential explanatory variables such as gender to enhance physiotherapists’ overall competence. For example, a study [50] showed that female physiotherapists exhibited greater confidence than their male counterparts in providing continued physiotherapy for patients with mental health conditions, highlighting the need for further investigation into such differences.

Aligned with the original PEAK qualitative study [31] exploring physiotherapists’ experiences with the training program, our interview findings also validate the perceived benefits for skill development and the reframing of beliefs regarding OA management. In addition, participants in our study provided several insights for improving the program to ensure that its effectiveness can be sustained in real clinical settings. Specifically, many participants highlighted the importance of adapting consultation videos and exercise demonstrations to an Asian context. This is supported by a scoping review [51] that emphasizes that cultural competencies can facilitate clinical, training, and administrative adjustments in mobile health. Furthermore, participants suggested incorporating more interactive modules with multimedia. Designs like the ATLAS (The Arthritis Training, Learning, and Up-Skilling for Health Professionals) e-learning program [52] that includes more interactive media functions could be considered to enhance engagement and understanding.

Our qualitative findings also underscore the need for additional practical training such as mock consultations. This aligns with previous evidence [53,54], which highlights the importance of practical components in health care education, particularly simulation experiences and structured supervision. To enhance telehealth training, future implementations of PEAK program could incorporate mock interviews where researchers play the role of patients and real patients, with detailed scripts guiding the consultation process. The scripts could also incorporate specific dialogues to address frequent challenges in telehealth communication such as maintaining patient engagement and explaining complex exercise plans over a screen. Additionally, these mock consultations should be recorded to allow for postsession review and feedback. Trainers or experienced professionals could provide guidance on improving communication, tone, and the handling of technological barriers during the telehealth consultation. To further enhance practical training, instructors could simulate a variety of telehealth scenarios, including technical difficulties such as poor internet connection, audio or video malfunctions, and patient difficulties with technology. These activities could also help physiotherapists build confidence in troubleshooting common telehealth issues while maintaining professionalism.

### Limitations

This study has several limitations. The majority of the participants were early-career practitioners holding entry-level certifications, which may limit the generalizability of the findings to more experienced professionals such as those with intermediate certifications or physician-level rehabilitation qualifications. Additionally, although our study demonstrated improvements in self-reported confidence and knowledge, it did not include objective assessments of real-world clinical performance such as simulated patient consultations to validate the practical application of learned skills. Moreover, although the PEAK program was culturally adapted, certain contents such as exercise videos featuring non-Asian models may have reduced its perceived relevance among participants. Lastly, as data were collected only immediately after training program, the long-term effects remain unknown. Further research is needed to assess sustained improvements in confidence and knowledge and whether this e-learning leads to real changes in their clinical practice. Overall, future studies should address these limitations to enhance the program’s applicability and impact.

### Conclusions

The PEAK-Chinese program significantly improved rehabilitation practitioners’ confidence and knowledge in managing knee OA, providing evidence of the potential for e-learning programs to support professional development and promote evidence-based OA care in China. Enhancing the cultural relevance of the content, addressing persistent misconceptions about OA in education, and incorporating practical, real-world training will further strengthen this program’s applicability in clinical settings.

## Appendix

Multimedia Appendix 1 CONSORT-EHEALTH (Consolidated Standards of Reporting Trials of Electronic and Mobile Health Applications and Online Telehealth; version 1.6) report.

Multimedia Appendix 2 Outcome measures.

## Abbreviations

ATLAS: Arthritis Training, Learning, and Up-Skilling for Health Professionals
CONSORT: Consolidated Standards of Reporting Trials
KOAKS: Knee Osteoarthritis Knowledge Scale
OA: osteoarthritis
PEAK: Physiotherapy Exercise and Physical Activity for Knee Osteoarthritis

## Data Availability

Requests for access to the data supporting this study can be directed to the corresponding author.
