# Supplementary material for: Effects of an e-Learning Program (Physiotherapy Exercise and Physical Activity for Knee Osteoarthritis [PEAK]) on Chinese Physical Therapists’ Confidence and Knowledge: Randomized Controlled Trial
Source: J Med Internet Res. 2025 Apr 18;27:e71057. doi: 10.2196/71057 (PMC12048791; doi:10.2196/71057)
Supplement: Multimedia Appendix 2 [file jmir_v27i1e71057_app2.docx]

**Outcome measures Appendix**

**Primary outcome 1- confidence in exercise therapy knowledge**

How confident are you in your knowledge about exercise to manage patients with knee OA?

0 1 2 3 4 5 6 7 8 9 10

|  |  |  |  |  |  |  |  |  |  |
| --- | --- | --- | --- | --- | --- | --- | --- | --- | --- |
|  |  |  |  |  |  |  |  |  |  |

Not at all confident Extremely confident

**Primary outcome 2- confidence in exercise program prescription**

How confident are you in your ability to prescribe a strengthening exercise program for patients with knee OA?

0 1 2 3 4 5 6 7 8 9 10

|  |  |  |  |  |  |  |  |  |  |
| --- | --- | --- | --- | --- | --- | --- | --- | --- | --- |
|  |  |  |  |  |  |  |  |  |  |

Not at all confident Extremely confident

**Secondary outcome 1- Knee Osteoarthritis Knowledge Scale**

Please rate each statement as:

☐ False ☐ Possibly False ☐ Unsure ☐ Possibly True ☐ True

- Your knee joint wears out with everyday use
- Osteoarthritis will only get worse over time
- Increased knee pain always means that you have damaged your knee
- You need an X-ray or scan to know if you have osteoarthritis
- Being active makes osteoarthritis feel better
- Keeping a healthy body weight is a key part of osteoarthritis care
- X-rays or scans show how much your osteoarthritis affects you
- Making your leg muscles stronger improves your ability to do daily tasks
- Pain from osteoarthritis can be managed without surgery
- Exercises can ease pain as much as most medications
- Most people with knee osteoarthritis will need a joint replacement at some point

**Secondary outcome 2-confidence in videoconference**

How confident are you in using videoconferencing to deliver care for patients with knee OA?

0 1 2 3 4 5 6 7 8 9 10

|  |  |  |  |  |  |  |  |  |  |
| --- | --- | --- | --- | --- | --- | --- | --- | --- | --- |
|  |  |  |  |  |  |  |  |  |  |

Not at all confident Extremely confident

**Secondary outcome 3- likelihood in use patient education**

How likely are you to use education in a treatment plan for patients with knee OA

0 1 2 3 4 5 6 7 8 9 10

|  |  |  |  |  |  |  |  |  |  |
| --- | --- | --- | --- | --- | --- | --- | --- | --- | --- |
|  |  |  |  |  |  |  |  |  |  |

Not at all likely Extremely likely

**Secondary outcome 4- likelihood in use strengthening exercise**

How likely are you to use strengthening exercise in a treatment plan for patients with knee OA

0 1 2 3 4 5 6 7 8 9 10

|  |  |  |  |  |  |  |  |  |  |
| --- | --- | --- | --- | --- | --- | --- | --- | --- | --- |
|  |  |  |  |  |  |  |  |  |  |

Not at all likely Extremely likely

**Secondary outcome 5- likelihood in use physical activity**

How likely are you to use physical activity in a treatment plan for patients with knee OA

0 1 2 3 4 5 6 7 8 9 10

|  |  |  |  |  |  |  |  |  |  |
| --- | --- | --- | --- | --- | --- | --- | --- | --- | --- |
|  |  |  |  |  |  |  |  |  |  |

Not at all likely Extremely likely

**Process measures for intervention group**

**1. Usefulness of overall course**

How useful did you find the course overall?

| Not at all useful | Somewhat useful | Moderately useful | Extremely useful |
| --- | --- | --- | --- |
|  |  |  |  |

**2. Usefulness of downloadable resources**

How useful did you find the downloadable resources?

| Not at all useful | Somewhat useful | Moderately useful | Extremely useful |
| --- | --- | --- | --- |
|  |  |  |  |

**3. Usefulness of video exercise library**

How useful did you find the video exercise library?

| Not at all useful | Somewhat useful | Moderately useful | Extremely useful |
| --- | --- | --- | --- |
|  |  |  |  |

**4. Usefulness of tele-health delivery**

How useful did you find the e-learning module in helping with videoconferencing consultations?

| Not at all useful | Somewhat useful | Moderately useful | Extremely useful |
| --- | --- | --- | --- |
|  |  |  |  |
